# Resveratrol Is a Natural Inhibitor of Human Intestinal Mast Cell Activation and Phosphorylation of Mitochondrial ERK1/2 and STAT3

**DOI:** 10.3390/ijms22147640

**Published:** 2021-07-16

**Authors:** Sabrina Bilotta, Lakshmi Bhargavi Paruchuru, Katharina Feilhauer, Jörg Köninger, Axel Lorentz

**Affiliations:** 1 Department of Nutritional Medicine, University of Hohenheim, Fruwirthstraße 12, 70593 Stuttgart, Germany; sabrina.bilotta@uni-hohenheim.de; 2 Department of Biochemistry and Molecular Biology, Institute for Medical Research—Israel-Canada, The Hebrew University of Jerusalem, Jerusalem 91120, Israel; bhargavi.lakshmi@mail.huji.ac.il; 3 Clinic for Visceral Surgery, Katharinenhospital, Kriegsbergstraße 60, 70174 Stuttgart, Germany; k.feilhauer@klinikum-stuttgart.de (K.F.); j.koeninger@klinikum-stuttgart.de (J.K.)

**Keywords:** mast cells, allergy, nutraceuticals, resveratrol, polyphenols, ERK1/2, STAT3, mitochondrial signaling

## Abstract

Mast cells play a critical role as main effector cells in allergic and other inflammatory diseases. Usage of anti-inflammatory nutraceuticals could be of interest for affected patients. Resveratrol, a natural polyphenol found in red grapes, is known for its positive properties. Here, we analyzed the effects of resveratrol on FcεRI-mediated activation of mature human mast cells isolated from intestinal tissue (hiMC). Resveratrol inhibited degranulation and expression of cytokines and chemokines such as CXCL8, CCL2, CCL3, CCL4, and TNF-α in a dose-dependent manner. Further, resveratrol inhibited the phosphorylation of extracellular signal-regulated kinase (ERK) 1/2 and signal transducer and activator of transcription (STAT) 3. ERK1/2 is known to be involved in cytokine expression of hiMC and to directly interact with STAT3. Mitochondrial STAT3 is phosphorylated by ERK1/2 and contributes to mast cell degranulation. We were able to isolate mitochondrial fractions from small hiMC numbers and could show that activation of mitochondrial STAT3 and ERK1/2 in hiMC was also inhibited by resveratrol. Our results indicate that resveratrol inhibits hiMC activation by inhibiting the phosphorylation of mitochondrial and nuclear ERK1/2 and STAT3, and it could be considered as an anti-inflammatory nutraceutical in the treatment of mast cell-associated diseases.

## 1. Introduction

Mast cells (MC) are key effector cells of type I allergic reactions; thus, they are closely related to allergic diseases, such as food allergies, as well as being linked to neuroimmune and inflammatory disorders, such as intestinal diseases [1,2,3]. Their main pro-inflammatory property is the release of inflammatory mediators such as pre-stored histamine or proteases, as well as de novo-synthesized cytokines or lipid mediators, after activation via diverse stimuli, of which the most important activation signal is the IgE-dependent stimulation of FcεRI IgE-receptor crosslinking by antigens [4]. The prevalence of allergies or intestinal diseases has increased in western countries in recent decades [5,6,7], thus simultaneously increasing patients’ need for specific pharmaceutic medication that is often associated with negative side effects [8]. Natural substances could be a good alternative or additive therapy, and they are associated with better compliance. In the context of so-called *nutraceuticals*, the polyphenol resveratrol could be of special interest because of its beneficial immunomodulatory effects [9].

Resveratrol (trans-3,4′,5 trihydroxystilbene, trans-Resveratrol) is mainly found in grapes, berries, or peanuts. In context of allergies, the polyphenolic compound was one amongst others that was able to prevent the development of a food allergy in mice [10], ameliorating the effects of atopic dermatitis and allergic rhinitis [11,12]. The anti-allergic and anti-inflammatory effects of resveratrol on different types of mast cell models have been shown previously [13,14,15]. Resveratrol suppressed IL-6 and TNF-α expression in mouse bone marrow-derived mast cells [13]. In the rat basophilic leukemia mast cell line (RBL-2H3), resveratrol was found to diminish β-hexosaminidase and histamine release [16]. Further, resveratrol was found to inhibit human eosinophil degranulation, as well as phosphorylation of protein kinases p38 and extracellular signal-regulated kinase 1/2 (ERK1/2) [17]. In children and adults with allergic rhinitis, intranasal administered resveratrol ameliorated clinical symptoms [18,19].

Degranulation of mast cells requires mitochondrial translocation to the site of exocytosis [20], suggesting the involvement of mitochondrial oxidative phosphorylation (OXPHOS) in mast cell exocytosis. In parallel, mitochondrial signal transducer and activator of transcription (STAT) 3 was found to be involved in ATP production by influencing the electron transport chain [21]. Moreover, STAT3 was shown and to be essential for immunologically mediated degranulation of human and mouse mast cells, as well as RBL-2H3 cells [22]. In IgE-antigen-activated RBL-2H3 cells, mitochondrial STAT3 was found to be phosphorylated by ERK1/2 on serine residue S727 [22]. Furthermore, we and others found that citrus flavonoids, especially nobiletin, are inhibitors of ERK1/2 and STAT3 [23,24] that downregulate mast cell degranulation, suggesting that nonpeptidic small molecules, such as polyphenols, are able to inhibit mast cells by downregulation of mitochondrial activity and the inhibition of ERK1/2 or STAT3.

The important role of signaling molecules such as ERK1/2 and STAT3 makes them potential targets for alternative natural-based medication, referred to as *nutraceuticals*, in the treatment of diseases involving mast cells. Here, we examine the immunomodulatory role of resveratrol on human intestinal mast cells (hiMC), as well as on the involved signaling molecules. We show that resveratrol strongly inhibits mast cell activation and downregulates phosphorylated ERK1/2 and STAT3 in mitochondrial fractions of hiMC.

## 2. Results

### 2.1. Resveratrol Has No Toxic Effect on hiMC and Inhibits Mast Cell Degranulation and Chemokine Expression in a Dose-Dependent Manner

**Figure 1:**
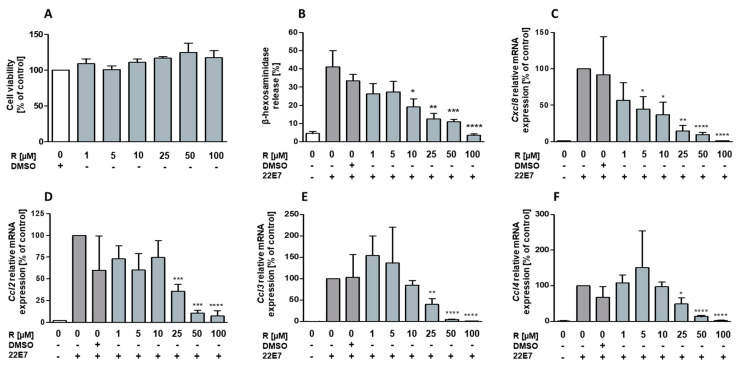
Cell viability, degranulation, and chemokine expression in human intestinal mast cells (hiMC) following treatment with resveratrol. Evaluation of cytotoxic effects of resveratrol on hiMC (**A**). Cells/well were incubated with 1, 5, 10, 25, 50, and 100 μM of resveratrol and the corresponding DMSO control for 24 h. After incubation, living cells in percent of the DMSO control is shown (*n* = 3). Release of β-hexosaminidase by hiMC (**B**) (*n* = 6) and mRNA expression of *Cxcl8* (**C**) (*n* = 4), *Ccl2* (**D**) (*n* = 6), *Ccl3* (**E**) (*n* = 6), and *Ccl4* (**F**) (*n* = 6). Cells were incubated with 1–100 μM of resveratrol for 60 min prior to stimulation by FcεRI crosslinking using 100 ng/mL of monoclonal antibody (mAb) 22E7 for 90 min. Controls were treated with the corresponding concentration of the vehicle DMSO. Values are mean ± SEM. * *p* < 0.05; ** *p* < 0.01; *** *p* < 0.001; **** *p* < 0.0001 compared to induced control treated with DMSO.

Mast cell activity is reported to be inconsistently affected by treatment with resveratrol [14,16,25,26] in several murine and human mast cell models. Thus, we aimed to investigate the effect of this polyphenol on primary mature human mast cells. We started to analyze whether resveratrol had any effect on cell viability of mast cells isolated from human intestinal tissue. HiMCs were incubated with 1–100 μM of resveratrol, concentrations previously used in mast cell models, for 24 h. Cell viability was determined by living cell counting and cytotoxicity was measured by absorbance of the MTT formazan product. Resveratrol did not show cytotoxic effects on cells when incubated for 24 h (Figure 1A, Supplemental Figure S1). To examine the effect of resveratrol on mediator release and gene expression, hiMCs were treated with 1–100 μM of resveratrol 1 h before stimulation via FcεRI crosslinking for 90 min. Degranulation, measured as β-hexosaminidase release, was found to be reduced by resveratrol in a dose-dependent manner. Thus, concentrations beginning at 10 μM significantly attenuated degranulation, showing the strongest effects at 100 μM (Figure 1B). To ascertain whether resveratrol also dose-dependently affected expression of de novo-synthesized mediators such as cytokines, we analyzed mRNA expression of the chemokine genes *Cxcl8*, *Ccl2*, *Ccl3*, and *Ccl4*. Treatment with concentrations of 5 μM of resveratrol or higher resulted in significant downregulation of *Cxcl8*, and *Ccl2*, *Ccl3*, and *Ccl4* were dose-dependently downregulated by concentrations of 25 μM and higher (Figure 1C–F).

### 2.2. Resveratrol and Nobiletin Act Similarly to STAT3 Inhibitor on Mast Cell Activity

**Figure 2:**
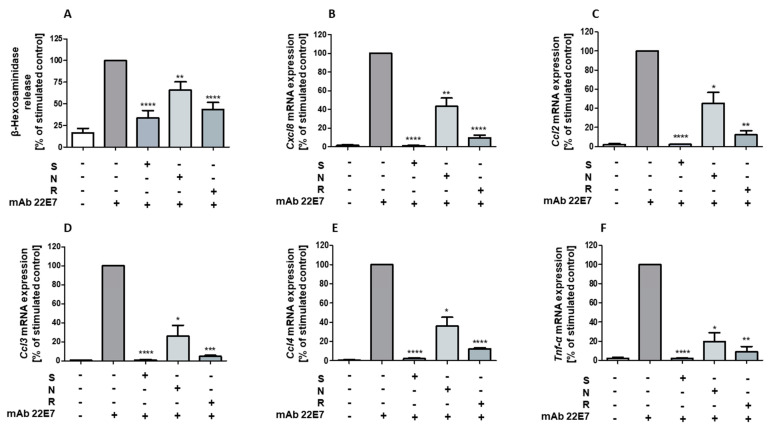
Degranulation and chemokine expression in hiMC following treatment with stattic, nobiletin, and resveratrol. Release of β-hexosaminidase (**A**) (*n* = 9) and mRNA expression of Cxcl8 (**B**) (*n* = 4), Ccl2 (**C**) (*n* = 3), Ccl3 (**D**) (*n* = 3), Ccl4 (**E**) (*n* = 3), and Tnf-α (**F**) (*n* = 3) by hiMC following treatment with 60 μM of stattic (S), 45 μM of nobiletin (N), or 50 μM of resveratrol (R), or corresponding concentrations of the vehicle DMSO (control). Cells were stimulated by FcεRI crosslinking using 100 ng/mL of mAb 22E7 for 5–90 min (**A**) and 90 min (**B**–**F**). Results are shown in % of stimulated control. Values are mean ± SEM. * *p* < 0.05; ** *p* < 0.01; *** *p* < 0.001; **** *p* < 0.0001.

Resveratrol and the citrus flavonoid nobiletin were able to inhibit mast cell degranulation [23]. Moreover, STAT3 in mitochondria was found to be an important molecule required for this process by directly influencing ATP production [22]. To investigate the inhibition of mast cells by polyphenols, we examined the effects of nobiletin and resveratrol in comparison to STAT3 inhibitor stattic on mast cell degranulation and chemokine mRNA expression in hiMC. We found that the FcεRI-mediated degranulation of mast cells was totally inhibited by treatment with stattic and almost totally inhibited by treatment with resveratrol. Treatment with nobiletin significantly reduced mast cell degranulation but to a lesser extent (Figure 2A). Additionally, expression of *Cxcl8*, *Ccl2*, *Ccl3*, *Ccl4*, and of *Tnf-α* were determined in response to treatment with stattic, nobiletin, and resveratrol before stimulation of mast cells by FcεRI crosslinking. As shown in Figure 2B–F, stattic and resveratrol inhibited the expression of all cytokines by almost 100% compared to the stimulated control in hiMC. Again, the effect of nobiletin was less pronounced.

### 2.3. Phosphorylation of STAT3 and ERK1/2 Is Diminished by Resveratrol and, to a Lesser Extent, by Nobiletin

**Figure 3:**
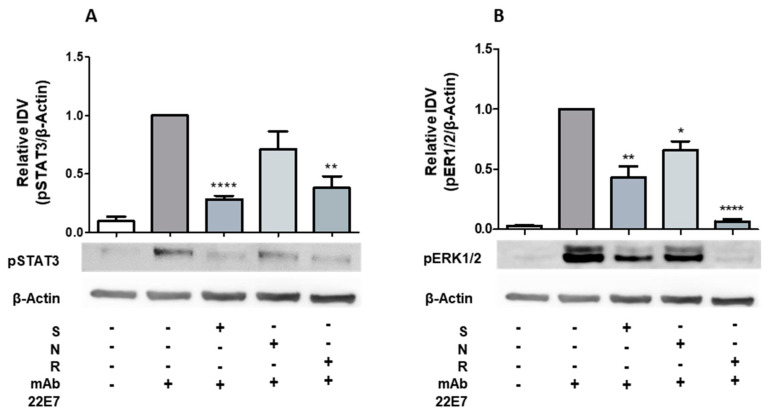
Phosphorylation of signal transducer and activator of transcription 3 (STAT3) and extracellular signal-regulated kinase 1/2 (ERK1/2) in hiMC following treatment with stattic, nobiletin, and resveratrol. Western blot analyses of (**A**) phosphorylated STAT3 (*n* = 5) and (**B**) phosphorylated ERK1/2 (*n* = 4) in hiMC. HiMCs were treated with 60 μM of stattic (S), 45 μM of nobiletin (N), 50 μM of resveratrol (R), or corresponding concentrations of the vehicle DMSO (control) before stimulation by FcεRI crosslinking using 100 ng/mL of mAb 22E7 for 5 min. Representative pictures and densitometric analyses in relation to stimulated control are shown. Values are mean ± SEM. * *p* < 0.05, ** *p* < 0.01, **** *p* < 0.0001.

Resveratrol and, to a lesser extent, nobiletin show inhibitory effects on degranulation and cytokine expression in hiMCs such as STAT3 inhibitor stattic. Thus, we next analyzed the effects of resveratrol and nobiletin on the phosphorylation of STAT3 in hiMC in response to FcεRI crosslinking. As expected, strong inhibition of phosphorylated STAT3 was achieved by STAT3 inhibitor stattic. Importantly, resveratrol also inhibited phosphorylation of STAT3. Treatment with nobiletin resulted in reduced phosphorylation, which was not significant (Figure 3A). It is already known that mitochondrial STAT3 is phosphorylated by ERK1/2 [22] and that ERK1/2 translocate to mitochondria, with an impact on the regulation of mitochondrial activity. Therefore, we examined the effects of resveratrol, nobiletin, and stattic on phosphorylation of ERK1/2 in hiMC following FcεRI crosslinking. Treatment with resveratrol results in almost complete inhibition of ERK1/2 activation. Additionally, inhibition by stattic and nobiletin was significant but not as strong as it was with resveratrol (Figure 3B).

### 2.4. Phosphorylated STAT3 and ERK1/2 Are Detectable in Mitochondrial Fractions of hiMC after FcεRI Crosslinking and Are Inhibited by Resveratrol

**Figure 4:**
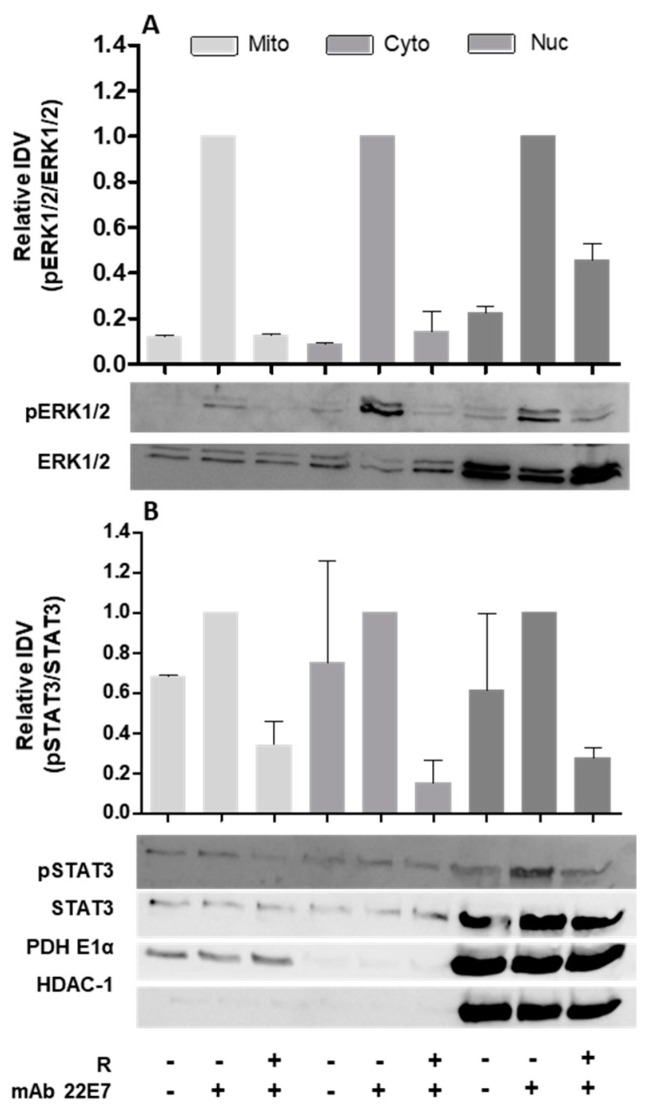
Phosphorylation of STAT3 and ERK1/2 in nuclear and mitochondrial fractions of hiMC following treatment with resveratrol. Western blot analyses of two independent experiments of (**A**) phosphorylated ERK1/2 and (**B**) phosphorylated STAT3 after subcellular fractionation in pure mitochondria (Mito), cytosol fraction (Cyto), and crude nuclear fraction (Nuc), respectively. HiMCs were treated with 50 μM of resveratrol (R), or corresponding concentrations of the vehicle DMSO (control), and stimulated by FcεRI crosslinking with 100 ng/mL of mAb 22E7 for 5 min. Exemplary pictures and densitometric analyses in relation to stimulated control for Western blots of phosphorylated ERK1/2 and total ERK1/2, and phosphorylated STAT3 and total STAT3, are shown. Values are mean ± SEM (*n* = 2).

Mast cell degranulation and mitochondrial activity were shown to be closely related [20]. Other than its canonical role, STAT3 is phosphorylated by ERK1/2 in mitochondrial fractions of RBL-2H3 cells [22], and it directly contributes to exocytosis. To prove that polyphenols directly affect phosphorylated STAT3 and ERK1/2 in mitochondria, these cell organelles had to be isolated from mast cells by subcellular fractionation. First, fractionation had to be optimized by reducing cell numbers because very high cell numbers, as suggested by available protocols, are not reachable using mature human mast cells from intestinal tissue [22,27,28]. PDH E1α was used as mitochondrial marker and HDAC-1 was used to show the nuclear fraction. It should be noted that the nuclear fraction also contains crude cell extract, which is not separated during fractionation. Having optimized the subcellular fractionation protocol for mitochondrial fractions down to 2 × 10^6^ hiMC per condition, we attempted to detect phosphorylated STAT3 and phosphorylated ERK1/2 in mitochondrial fractions. We were able to detect phosphorylated ERK1/2 (Figure 4A) and phosphorylated STAT3 (Figure 4B) in mitochondrial fractions of hiMC. More importantly, we found that treatment of hiMC with resveratrol before stimulation by FcεRI crosslinking results in the inhibition of activated ERK1/2 and STAT3 phosphorylation in mitochondrial fractions of hiMC.

## 3. Discussion

In the present study, we show that resveratrol is a potent inhibitor of mature human mast cell activation. Resveratrol shows strong inhibitory effects on the release of pre-stored mediators and the expression of de novo-synthesized mediators. Further, we could show that resveratrol inhibits activation of ERK1/2 and STAT3 in both nuclear and mitochondrial fractions of hiMC. Resveratrol shows stronger inhibitory effects, e.g., compared to nobiletin, a polymethoxyflavone from citrus peel. Thus, resveratrol might be a potential natural medication alternative, referred to as *nutraceutical*, in the treatment of mast cell-associated diseases, such as allergies.

The effect of polyphenols such as resveratrol in relation to IgE-dependent MC activation has been analyzed in different mast cell models. As such, resveratrol was found to potently inhibit mast cell degranulation in RBL-2H3 cells (up to 50%) [16,25] and mouse BMMC (50%) [26]. In primary human skin mast cells, degranulation was not affected by low concentrations of resveratrol (<50 μM), but only in the range of 50–100 μM. We found inhibition of degranulation in hiMC starting at 10 μM, with the strongest effect up to almost 100% at 100 μM of resveratrol. In contrast to human skin mast cells, we did not detect enhanced expression of TNF-α in hiMC following treatment with low concentrations of resveratrol (<10 μM) (not shown) [15]. Resveratrol strongly decreased expression of *Cxcl8*, *Ccl2*, *Ccl3*, *Ccl4*, and *Tnf-α* in hiMC. In human mast cell line HMC-1, picetannol, a resveratrol metabolite, was also able to reduce the gene expression of *Tnf-α* and *Cxcl8* [29].

Several chemokines are increased in inflammatory processes such as allergies [30]. CCL2, CCL3, and CCL4 are regulatory factors in immune and endothelial regulation, as well as in chemotaxis [31]. It was previously shown that CCL2 recruits macrophages to sites of inflammation after allergen exposure [32], and that the blocking of CCL2 signaling pathway prevents Th2 inflammatory response in allergic asthma [33]. TNF-α and CXCL8 were reported to serve as important inflammatory cytokines by attracting neutrophils and basophils and promoting inflammatory reactions, not only in relation to allergies [34,35,36].

The induction of CCL2 and CXCL8 is regulated by the MAPK signaling pathway. It was found that the application of ERK-specific inhibitors on human eosinophils reduced the release of CCL2 and CXCL8 [37]. In the human MC line LAD2, and in human cord blood-derived MC, expression and release of CCL2 and CCL5 was induced by IL-33; this induction was due to the activation of the MAPK signaling pathway, even though ERK showed no direct influence on chemokine expression for these cells [38]. In LAD2 cells, CCL2 production was induced by C3a complement component-dependent MC activation, which was shown to be inhibited by usage of the U0126 inhibitor of MEK-induced ERK phosphorylation [39]. In contrast, in IgE-activated RBL-2H3 cells, CCL2 production was not affected by ERK1/2 inhibition [40]. In hiMC, the MAPK family is well known to be involved in cytokine expression [23,41,42,43]. In addition, we found that flavonoids nobiletin and, to a lesser extent, tangeritin show inhibitory effects on ERK1/2 phosphorylation, as well as on *Cxcl8*, *Ccl3*, and *Ccl4* expression [23]. Here, we show that resveratrol inhibits FcεRI-mediated phosphorylation of ERK1/2 and the expressions of *Cxcl8*, *Ccl2*, *Ccl3*, and *Ccl4* in hiMC. Inhibition of ERK1/2 phosphorylation by resveratrol was also found in HMC-1 cells and at a high concentration in human skin mast cells [14,15]. However, it should be noted that the inhibitory effect of resveratrol on ERK1/2 was very pronounced, but not limited to it. Phosphorylation of other IgE-dependently activated kinases, such as Akt or JNK in hiMC (Supplemental Figure S2), or Akt and p38 in human skin mast cells, was also reduced in response to treatment with resveratrol [15].

MAP kinase ERK1/2 is further known to directly affect STAT3 [22], which in turn is involved in ATP production in mitochondria. It is known that MC degranulation requires mitochondrial translocation to sites of exocytosis and that OXPHOS may be a central process in MC activation [20]. We therefore tested whether resveratrol affects phosphorylation of ERK1/2 and STAT3. Indeed, resveratrol displays inhibitory effects on both molecules. Aside from its canonical role as a transcription factor, STAT3 was shown to participate in electron transport in the process of OXPHOS-dependent ATP production in mitochondria [21]. ATP serves as an energy source for MC degranulation. Two STAT3 inhibitors, mitocur-1 and -3 based on curcumin, were able to affect degranulation and cytokine release in murine and primary human mast cells, and diminished ATP levels in cells cultured in glucose-free medium, indicating a direct effect on mitochondrial ATP production. Additionally, both curcumin-based inhibitors decreased histamine release in acute anaphylaxis in mice [44]. Curcumin is a polyphenol obtained from turmeric and is intensively discussed as alternative medication due to its positive biological properties [45]. STAT3 inhibitors mitocur-1 and mitocur-3 are directed against mitochondrial STAT3, reducing its phosphorylation on serine 727 residue. A resveratrol–caffeic acid hybrid was detected to affect and inhibit acetylation, as well as the phosphorylation of STAT3 on tyrosine residue T705, in two human cancer cell lines [46]. In hiMC, resveratrol was able to inhibit the activation of STAT3-S727 in both nuclear and mitochondrial fractions.

Isolation of hiMC from surgical tissue does not provide large cell numbers. Thus, to analyze mitochondrial fractions from mature hiMC, we had to optimize the fractionation for comparatively low cell numbers. Fortunately, we were able to isolate mitochondrial fractions from small hiMC numbers and could show that phosphorylated ERK1/2 and STAT3 were present in this fraction. Resveratrol was found to inhibit the phosphorylation of ERK1/2 and STAT3 in mitochondria of hiMC activated via crosslinking of the FcεRI receptor. Occurrence and activation of ERK1/2 and STAT3 in mitochondria suggest the importance of signaling molecules present in mitochondria in terms of MC activity.

The increase in MC-associated diseases requires novel treatment possibilities. Notably, negative side effects related to conventional medication may be overcome with natural-based alternatives, thus increasing patients’ acceptance. We have previously shown that plant-derived substances have the potential to inhibit the release of MC-specific mediators in hiMC. Cinnamon extract could reduce degranulation down to 20% in hiMC after IgE-dependent activation, as well as completely inhibit the expression of *Cxcl8*, *Ccl2*, *Ccl3*, *Ccl4*, and *Tnf-α* [23,42]. Cinnamaldehyde was thereby shown to be the active compound of cinnamon extract, leading to its anti-inflammatory actions in hiMC [47]. However, citrus flavonoids, such as nobiletin and tangeritin or stilbene resveratrol, may be more acceptable to patients than cinnamon extract or cinnamaldehyde. We found that resveratrol shows stronger inhibitory effects on hiMC than citrus flavonoids. *Citrus tachibana* leaf extract, with its components nobiletin and tangeritin, improved OVA-induced allergic symptoms such as diarrhea and rectal temperature [48]. Application of resveratrol for 13 days improved the same parameters in OVA-treated mice and reduced histamine and MC protease 1 in serum [16]. These observations show that inflammatory disorders associated with MC can be alleviated with natural occurring plant substances, and that resveratrol can be a highly potent anti-allergic plant substance.

In summary, our results show a strong inhibitory effect of resveratrol on hiMC degranulation and chemokine expression. These effects seem to be mediated by inhibition of ERK1/2 and STAT3. The data suggest that resveratrol could be considered as a potential natural-based anti-allergic component, a *nutraceutical*, for the treatment of MC-associated disorders such as allergies.

## 4. Materials and Methods

### 4.1. Isolation and Culture of hiMC

HiMCs were isolated from surgical tissue from patients who underwent bowel resection, as previously described [49]. Permission to conduct the study was obtained by the local ethical committee. Tissue underwent mechanical shredding and enzymatic digestion with pronase (Serva, Heidelberg, Germany) and collagenase D (Nordmark Biochemicals, Uetersen, Germany). After overnight culture of obtained cell suspension in RPMI 1640+GlutaMax^TM^ (Gibco Invitrogen, Paisley, OR, USA) with 10% FBS (Merck, Darmstadt, Germany), 100 μg/mL streptomycin, 100 U/mL penicillin (HyClone^TM^ Laboratories, South Logan, Utah, USA), 100 μg/mL gentamycin, and 2.5 μg/mL amphotericin B (CarlRoth Karlsruhe, Germany), enrichment of cells by magnetic cell separation of c-Kit+ (CD117) cells was conducted using *CD117 microbead kit* after *dead cell removal kit* (MACS^TM^ system, Miltenyi Biotech, Bergisch Gladbach, Germany). Pure hiMCs were cultured with 25 ng/mL stem cell factor (SCF) (PeproTech, Hamburg, Germany) and 2 ng/mL IL-4 (PeproTech) for at least 14 days before use in experiments.

### 4.2. Cell Viability

Next, 5 × 10^4^ hiMCs per well were incubated in a 48-well plate in the presence of 1, 5, 10, 25, 50, or 100 μM resveratrol (Merck, Darmstadt, Germany), respectively, or the vehicle DMSO (CarlRoth). After 24 h, living cells were counted after trypan blue staining. Additionally, an MTT assay was performed. For that, 25 μL of MTT solvent (Merck) was added to each well and incubated for 3 h. Supernatant was discarded, 100 μL of lysis solution was added to each well and gently mixed, and absorbance of MTT formazan product was measured to detect the amount of substrate converted by living cells.

### 4.3. Treatment of hiMC

Cells were treated with 1, 5, 10, 25, 50, or 100 μM resveratrol or 45 μM nobiletin (Indofine Chemical, Hillsborough, NJ, USA) 1 h prior to stimulation by FcεRI crosslinking using 100 ng/mL monoclonal antibody (mAb) 22E7 directed against the FcεRI α-chain (Genentech, South Francisco, CA, USA). Cells were stimulated for 5 or 90 min to analyze degranulation, for 90 min to analyze mRNA expression, and for 5 min to detect activated signaling molecules. To analyze STAT3 activation, hiMCs were pre-incubated for 20 min with 60 μM STAT3 inhibitor stattic (Merck) prior to stimulation by FcεRI crosslinking. Unstimulated controls contained the same concentrations of vehicle DMSO.

### 4.4. Degranulation

Degranulation of MC was measured by determining the amount of released β-hexosaminidase in supernatants by a color enzyme assay [50].

### 4.5. RNA Preparation and Real-Time RT-PCR

Total RNA was obtained by using an EXTRACTME TOTAL RNA kit (blirt, Gdansk, Poland). Real-time RT-PCR reactions were performed in optical tubes containing 1 μL of cDNA template, 0.125 μL each sense and anti-sense primer, 4 μL of H_2_O, and 5 μL of SsoFast^TM^ EVAGreen Supermix (Bio-Rad Laboratories, Feldkirchen, Germany). Reaction mixture without cDNA was used as negative control. Relative quantification (2^−ΔΔCt^) was performed using glyceraldehyde 3-phosphate dehydrogenase (*Gapdh*) housekeeping gene as reference. Sense and antisense primer sequences were: *Gapdh*: 5′-TGG TCT CCT CTG ACT TCA AC-3′, 5′-CCT GTT GCT GTA GCC AAA TT-3′, product size: 128 bp; *Cxcl8*: 5′-CTG AGA GTG ATT GAG AGT GG-3′, 5′-ACA ACC CTC TGC ACC CAG TT-3′, product size: 113 bp; *Ccl2*: 5′-CTT CTG TGC CTG CTG CTC AT-3′, 5′-CGG AGT TTG GGT TTG CTT GTC-3′, product size: 273 bp; *Ccl3*: 5′-CTC TGC ATC ACT TGC TGC TGA CAC-3′, 5′- CAC TCA GCT CCA GGT CGC TGA C-3′, product size: 212 bp; *Ccl4*: 5′- GCT AGT AGC TGC CTT CTG CTC TCC-3′, 5′-CAG TTC CAG CTG ATA CAC GTA CTC C-3′, product size: 238 bp; *Tnf-α*: 5′-CAG ATA GAT GGG CTC ATA CCA GGG-3′, 5′-GCC CTC TGG CCC AGG CAG TCA G-3′, product size: 377 bp (all Eurofins, Ebersberg, Germany). CFX 2.1 software and a CFX Connect Real-Time PCR System of Bio-Rad Laboratories were used.

### 4.6. Isolation of Mitochondria from hiMC

Subcellular fractionation protocol for the purification of hiMC mitochondrial fractions was adapted from Sharkia et al. [51] and modified for isolation working with low cell numbers of 2–5 × 10^6^. Cell compartments were fractionated into mitochondria, nucleus, and cytosol by several ascending centrifugation and mechanical cell lysis using a syringe needle before lysis with RIPA buffer (0.01 mol/L Tris-Hcl, 1% deoxycholate, 0.1% SDS, 0.15 mol/L sodium chloride, 0.25 μmol/L phenylmethylsulfonylfluoride (all CarlRoth), and 1% Triton-X 100 (Merck)) containing protease inhibitor cocktail cOmplete^TM^ Ultra Tablets Mini and phosphatase inhibitors phosSTOP^TM^ (both Roche Diagnostics, Mannheim, Germany) and subsequent sonication. In brief, cell suspension was homogenized in buffer A (250 mM sucrose, 20 mM HEPES, 10 mM potassium chloride, 1.5 mM magnesium chloride, 1 mM EDTA, 1 mM EGTA (all CarlRoth), and 1 mM DTT (Invitrogen, Karlsruhe, Germany)) and passed through a syringe needle 10 times before centrifugation at 720× *g* for 5 min and 2000× *g* for 3 min. The obtained pellet was again homogenized with buffer A, pulled through a syringe needle 10 times, and centrifuged at 2000× *g* for 10 min before pellet lysis with RIPA buffer and sonication to obtain the nuclear fraction. Obtained supernatant from the first step was transferred to a clean tube and further centrifuged at 12,000× *g* for 10 min; obtained supernatant was marked as cytosol fraction and pellet homogenized with buffer A, and was pulled through a syringe 10 times before being centrifuged at 12,000× *g* for 10 min again. Mitochondrial fraction was lysed in RIPA buffer and by final sonication. All steps were performed at 4 °C.

### 4.7. Western Blot Analysis

Whole cell lysates were obtained by lysis of cells with extraction buffer containing 25 mM Tris pH7.4, 0.5 mM EDTA, 10 mM β-Mercaptoethanol (CarlRoth), and 0.05% Triton-X (Merck) supplemented with protease inhibitor cocktail cOmplete^TM^ Ultra Tablets Mini and phosphatase inhibitors phosSTOP^TM^ (both Roche Diagnostics). Whole cell lysates or subcellular fractions were separated in a 12% SDS-polyacrylamide gel and blotted onto nitrocellulose membrane (Immobilon^®^-P, CarlRoth) in 38.6 mmol/L glycine, 47.9 mmol/L tris base, 1.28 mmol/L SDS, and 20% methanol (CarlRoth, respectively) by blotting with Trans-Blot Cell (Bio-Rad). Membranes were blocked with 5% FBS in tris-buffered saline containing 0.1% Tween-20 (TBS-T) (CarlRoth) for at least 30 min at room temperature. Afterwards, membranes were probed with respective antibodies for phospho-STAT3 (S727), STAT3 (124H6), phospho-ERK1/2 (P44/42 MAPK, 137F5), HDAC-1 (D5C6U), phospho-Akt (4060s), phospho-SAP/JNK (2821) (Cell Signaling Technology^®^, Frankfurt, Germany), ERK1/2 (12D4) (Enzo^®^Life Sciences, Lausen, Switzerland), PDH-E1α (proteintech, St. Leon-Rot, Germany), or β-Actin (13E5) rabbit mAb (Cell Signaling Technology^®^) overnight at 4 °C, and the next day with respective HRP-linked secondary antibodies anti-mouse IgG or anti-rabbit IgG (Cell Signaling Technology^®^) for 60 min at room temperature. Visualization was performed by using SuperSignal^TM^ West Duration Substrate (ThermoFisher Scientific, Bonn, Germany) and an electro-chemiluminescence detection system (FluorChem; Biozym Scientific, Hessisch Oldendorf, Germany). Signals were measured by a bioimaging analyzer (Alpha Innotech Corporation, San Leandro, CA, USA) and normalization was performed with β-Actin or the corresponding unphosphorylated signal molecule. For detection of several proteins, membranes were stripped in 25 mM glycin and 1% SDS in a water bath at 37 °C, and probed again with the respective antibody.

### 4.8. Statistics

Data are expressed as mean ± standard error of the mean (SEM). Student’s *t*-test was used to analyze differences between two groups. GraphPad Prism scientific software version 5.0 (San Diego, CA, USA) was used for statistical analysis. Values of *p* < 0.05 were considered to be statistically significant.

## Supplementary Materials

The following are available online at https://www.mdpi.com/article/10.3390/ijms22147640/s1.
