# Supplementary material for: Resveratrol Is a Natural Inhibitor of Human Intestinal Mast Cell Activation and Phosphorylation of Mitochondrial ERK1/2 and STAT3
Source: Int J Mol Sci. 2021 Jul 16;22(14):7640. doi: 10.3390/ijms22147640 (PMC8307672; doi:10.3390/ijms22147640)
Supplement: Supplementary file 1 [file ijms-22-07640-s001.zip › ijms-1281099-supplementary.pdf]

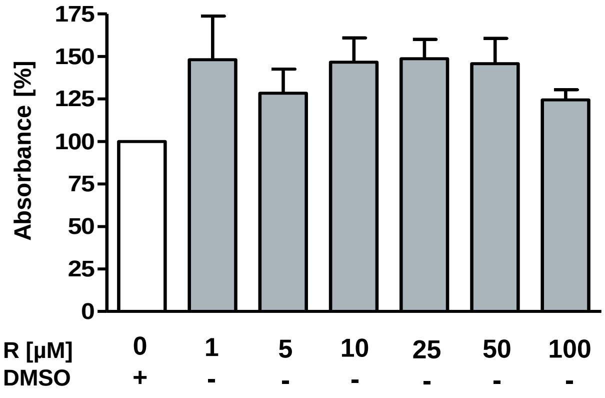

Figure S1. *Cell viability determined by MTT assay of human intestinal mast cells (hiMC) following treatment with resveratrol*. Cells were incubated with 1, 5, 10, 25, 50 and 100 µM resveratrol and the corresponding DMSO control for 24h. After incubation, MTT assay was conducted. Absorbance in percent of the DMSO control is shown (n = 3).

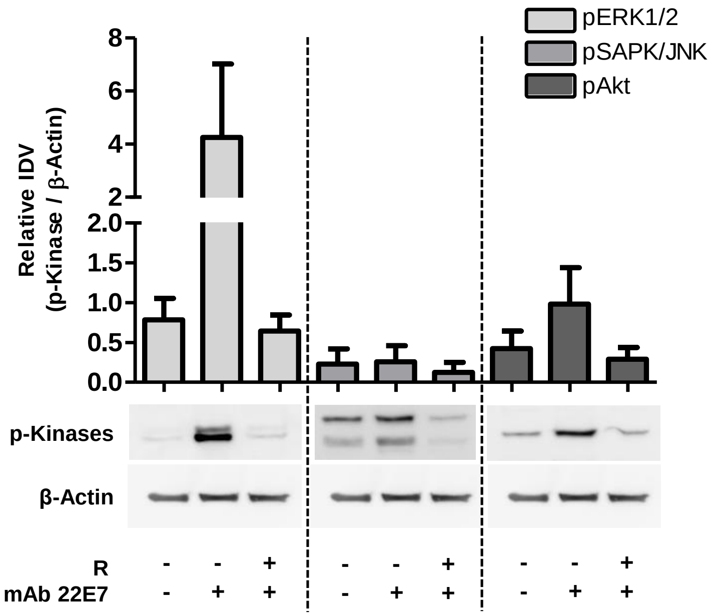

Figure S2. *IgE-mediated phosphorylation of extracellular signal-regulated kinase (ERK1/2), stress-activated protein kinases (SAPK)/Jun amino-terminal kinases (JNK) and protein kinase B (Akt) in hiMC following treatment with resveratrol.* Western Blot analyses of phosphorylated ERK1/2, phosphorylated SAPK/JNK and phosphorylated Akt in hiMC (n = 2). HiMC were treated with 50 μM resveratrol (R) or corresponding concentration of vehicle DMSO (control) before stimulation by FcεRI cross-linking using 100 ng/ml mAb 22E7 for 5 min. Representative pictures and densitometric analyses in relation to β-actin are shown. Values are mean ± SEM.
